# Effect of Epoxide Content on the Vulcanizate Structure of Silica-Filled Epoxidized Natural Rubber (ENR) Compounds

**DOI:** 10.3390/polym13111862

**Published:** 2021-06-03

**Authors:** Gyeongchan Ryu, Donghyuk Kim, Sanghoon Song, Kiwon Hwang, Byungkyu Ahn, Wonho Kim

**Affiliations:** 1 School of Chemical Engineering, Pusan National University, Busan 46241, Korea; 60chan95@gmail.com (G.R.); ehdgurzxc@gmail.com (D.K.); thdtkd1111@gmail.com (S.S.); kiwon8348@gmail.com (K.H.); 2 Hankook Tire & Technology Co., Ltd., R&D Center, 50 Yuseong-daero 935 beon-gil, Yuseong-gu, Daejeon 34127, Korea; bkahn8855@gmail.com

**Keywords:** truck bus radial tires, epoxidized natural rubber, silica-filled compound, vulcanizate structures, abrasion resistance

## Abstract

The demand for truck–bus radial (TBR) tires with enhanced fuel efficiency has grown in recent years. Many studies have investigated silica-filled natural rubber (NR) compounds to address these needs. However, silica-filled compounds offer inferior abrasion resistance compared to carbon black-filled compounds. Further, the use of NR as a base rubber can hinder silanization and coupling reactions due to interference by proteins and lipids. Improved silica dispersion be achieved without the use of a silane coupling agent by introducing epoxide groups to NR, which serve as silica-affinitive functional groups. Furthermore, the coupling reaction can be promoted by facilitating chemical interaction between the hydroxyl group of silica and the added epoxide groups. Thus, this study evaluated the properties of commercialized NR, ENR-25, and ENR-50 compounds with or without an added silane coupling agent, and the filler–rubber interaction was quantitatively calculated using vulcanizate structure analysis. The increased epoxide content, when the silane coupling agent was not used, improved silica dispersion, abrasion resistance, fuel efficiency, and wet grip. Once a basic level of silica dispersion was secured by using the silane coupling agent, both the abrasion resistance and wet grip improved with increasing epoxide content. Furthermore, the silane coupling agent could be partially replaced by ENR due to the high filler–rubber interaction between the ENR and silica. Therefore, epoxidation shows potential for resolving the issues associated with poor coupling reactions and abrasion resistance in silica-filled NR compounds.

## 1. Introduction

The fuel efficiency and abrasion resistance offered by truck bus radial (TBR) tires have had to improve over recent years to meet strengthened environmental regulations and the requirements of newly developed hydrogen fuel cell trucks [1,2]. Tire performance is significantly affected by the contact between the tire tread and the ground; thus the continued development of tread compounds is important for advancing the performance of tires. Synthetic rubber is often used as the base rubber for passenger car radial (PCR) tire treads. Therefore, efforts to improving tire performance have focused on adjusting the molecular weight, microstructure of synthetic rubber, and introducing functional groups [3,4]. However, TBR tire treads need to withstand a larger load and longer operation times. Thus, natural rubber (NR) is preferred as a base rubber to achieve superior mechanical properties and resistance to cut and chip. Accordingly, research regarding base rubbers for TBR tire treads remains limited compared to that on PCR tire treads.

Carbon black is compatible with NR, and has been used as a reinforcing filler when NR is used as the base rubber in TBR tires. Various studies have investigated the partial replacement of carbon black with silica to improve the fuel efficiency of TBR tires [5,6]. The use of silica with a silane coupling agent, such as bis-[3-(triethoxysilyl)propyl]-tetrasulfide (TESPT), leads to partial hydrophobization of the hydrophilic groups on the silica surface. This can promote the dispersion of silica within the rubber matrix, and is also known to give superior fuel efficiency compared to tires based on carbon black due to chemical reactions with the rubber chain [7]. However, the combination of silica with a silane coupling agent can lead to lower abrasion resistance of the compounds compared to carbon black [8]. Further, silica-filled NR compounds are associated with poor silica silanization and coupling reactions between rubber and the silane coupling agent due to the large molecular structure of NR and the influence of proteins and lipids [9,10].

These problems can be resolved by introducing epoxide groups to NR to serve as silica-friendly functional groups [10]. These epoxide groups can improve silica dispersion, and help to overcome the coupling issues associated with NR by encouraging filler–rubber interactions via chemical interactions between the NR epoxide groups and silica hydroxyl groups [11,12]. The reaction of formic acid and hydrogen peroxide with NR molecules leads to the formation of epoxidized natural rubber (ENR) in an NR latex state, as reported by Gelling [13], Perera [14], and Ng [15]. Commercialized ENR products include ENR-25 and ENR-50, which offer 25 mol% and 50 mol% epoxide group contents, respectively. Both ENR-25 and ENR-50 have been used to achieve high filler–rubber interactions via the epoxide group without the use of a silane coupling agent [16]. Moreover, some studies have reported that filler–rubber interactions are improved at higher epoxide contents when NR is partially replaced with ENR [17,18]. However, these previous studies evaluated filler–rubber interactions based on simple comparisons of the modulus of the stress–strain curve, or using bound rubber measurements. Thus, no quantitative analysis of the complex vulcanizate structures of silica-filled ENR compounds has yet been conducted.

Filler dispersion and filler–rubber interactions within the rubber have a significant effect on the mechanical properties of rubber vulcanizates, while crosslinking with sulfur can also play a role [19]. Thus, rubber vulcanizates with varying equilibrium swelling ratios in organic solvents have been previously analyzed according to the degree of the crosslinking network. For example, Flory and Rehner computed and formulated the crosslink density of unfilled vulcanizates based on this swelling phenomenon [20,21]. Further, in the filled vulcanizates, Kraus [22] and Boonstra [23] confirmed that the addition of an adhering filler, such as carbon black, led to restricted swelling behavior and changes in the total crosslink density. The Kraus equation was proposed based on this work, where the density was found to be proportional to the filler volume fraction. Furthermore, recent studies quantitatively distinguished the vulcanizate structure of compounds with silica as a filler as networks of silica–silane–rubber and sulfur chemical crosslinking [19,24,25,26,27,28,29,30,31,32]. Consequently, the filler–rubber network of silica-filled compounds can be quantitatively differentiated, the detailed vulcanizate structure of rubber with functional groups (e.g., ENR) can be identified, and the effects of functional groups on the properties of the compounds were better understood.

This study aimed to use vulcanizate structure analysis to determine the vulcanizate structure depending on the epoxide content of silica-filled compounds with NR, ENR-25, and ENR-50 as base rubbers. The effects of epoxide content on the properties of the compounds were also examined.

## 2. Materials and Methods

### 2.1. Materials

NR (Standard Vietnamese Rubber SVR-10, dirty content = 0.1 wt%, Astlett Rubber Inc., Oakville, ON, Canada), ENR-25, and ENR-50 (Epoxyprene 25, Epoxyprene 50, Muang Mai Guthrie Public Co., Ltd., Muang, Phucket, Thailand) were used as base rubbers. Silica (Ultrasil 7000 GR, Evonik Industries AG, Essen, Germany) with a Brunauer–Emmett–Teller (BET) surface area of 160 to 175 m^2^/g was used as a filler. Bis-[3-(triethoxysilyl)propyl]-tetrasulfide (TESPT, Si-69, Evonik Korea Ltd., Seoul, Korea) was used as the silane coupling agent. ZnO and stearic acid (Sigma-Aldrich Corp., Seoul, Korea) were used as activators, and N-(1,3-dimethyl butyl)-N-phenyl-p-phenylenediamine (6PPD, Kumho Petrochemical Co., Daejeon, Korea) was used as the antioxidant. Sulfur (Daejung Chemicals and Metals Co., Siheung, Korea) was used as the crosslinking agent. N-cyclohexyl benzothiazyl-2-sulfenamide (CBS, 98%, Tokyo Chemical Industry Co., Ltd., Tokyo, Japan) and 1,3-diphenylguanidine (DPG, 98%, Tokyo Chemical Industry Co., Ltd., Tokyo, Japan) were used as cure accelerators. N-cyclohexylthio phthalimide (PVI, Shandong Yanggu Huatai Chemical Co., Ltd., Liaocheng, China) was used as the pre-vulcanization inhibitor.

### 2.2. Manufacture of Compounds and Vulcanizates

Compounds were manufactured using an internal mixer (300cc, Mirae Scientific Instruments Inc., Gwangju, Korea) based on the formulations presented in Table 1. The fill factor was 80% of the mixer volume. The input unit was parts per hundred rubber (phr), and the compounds were proportional to the amount of rubber added. The effect of substituting the coupling agent TESPT using the epoxide groups in ENR was evaluated by comparing the compounds without TESPT with the compounds with 8 wt% TESPT with respect to silica.

The mixing procedure is outlined in Table 2, where the initial temperatures of the first and second stages were 100 and 50 °C, respectively, and the dump temperature ranges were 150 to 155 °C and 80 to 90 °C, respectively. After mixing was complete in each stage, the compounds were formed into sheets using a two-roll mill. The vulcanizates were prepared by pressing the compound in a hydraulic press at 150 °C for an optimal curing time (t_90_).

### 2.3. Characterization

#### 2.3.1. Proton Nuclear Magnetic Resonance Spectroscopy (^1^H NMR)

The epoxide content of the ENR samples was determined using proton nuclear magnetic resonance spectroscopy (^1^H NMR; Varian, Unity Plus 300, Garden State Scientific, Morristown, NJ, USA). Solutions of ENR (15 mg/mL) in deuterochloroform (CDCl_3_, Cambridge Isotope Laboratories, Inc., Andover, MA, USA) were prepared in 5-mm NMR tubes.

#### 2.3.2. Differential Scanning Calorimetry (DSC)

The glass transition temperature (T_g_) of the rubber was measured using differential scanning calorimetry (DSC-Q10, TA Instruments, USA). Rubber samples (3–6 mg) were measured from −80 to 100 °C at a heating rate of 10 °C/min.

#### 2.3.3. Payne Effect

The filler–filler interaction of the compound samples were determined using a rubber processing analyzer (RPA2000, Alpha Technologies, Hudson, OH, USA) according to the standard procedure given in ASTM D8059. The storage modulus (G′) of the compounds after the 1st stage of mixing was measured at 60 °C within a strain range of 0.01% to 40.04%. The storage modulus was high in the low strain region because silica agglomerates were not yet disintegrated, and decreased once higher strain was applied. The change in storage modulus (ΔG′) was calculated by subtracting the value at a strain of 40.04% from that at 0.28%. This value describes the Payne effect, and represents the degree of filler–filler interaction. Thus, it was used as an indicator for the degree of filler dispersion within the rubber compounds. The compounds were annealed for 5 min at 160 °C in the rubber processing analyzer, and the degree of re-agglomeration of silica was evaluated based on the Payne effect of the sample after annealing.

#### 2.3.4. Mooney Viscosity

The processability of the rubber was evaluated using the standard procedure given in ASTM D164. A Mooney viscometer (Vluchem IND Co., Seoul, Korea) was used to measure the torque as the rotor rotated at 2 rpm within a space filled with unvulcanized rubber. The sample was preheated to 100 °C over 1 min, and the torque was measured as the rotor was rotated for 4 min.

#### 2.3.5. Bound Rubber Content

After the first mixing stage, a sample of the compounds (0.2 ± 0.01 g) was placed on a filter paper and immersed in toluene (100 mL) for 6 days at 25 °C to extract the unbound rubber. The toluene containing the extracted unbound rubber was cleaned with acetone and dried. The bound rubber content was computed based on the sample weights before and after the experiment as follows:

(1) $$Bound\;rubber\;content\;\left(\%\right)=\frac{w_{fg}-w_{t}\left[\frac{m_{f}}{m_{f}+m_{r}}\right]}{w_{t}\left[\frac{m_{r}}{m_{f}+m_{r}}\right]}\times 100,$$

where *w_fg_* is the weight of the filler and gel, *w_t_* is the weight of the specimen, *m_f_* is the weight fraction of the filler in the compounds, and *m_r_* is the weight fraction of the polymer in the compounds.

#### 2.3.6. Cure Characteristics

The cure characteristics of the compounds were evaluated based on the minimum torque (T_min_), maximum torque (T_max_), scorch time (t_10_), and optimal cure time (t_90_) measured using a moving die rheometer (MDR, Myung Ji Co., Seoul, Korea) over 30 min at 150 °C and a vibration angle of ±1°.

#### 2.3.7. Crosslink Density and Vulcanizate Structure Analysis

Vulcanizate specimens (10 mm × 10 mm × 2 mm) were sequentially immersed in tetrahydrofuran (THF, 99%, Samchun Chemical Co., Seoul, Korea) and n-hexane (95%, Samchun Chemical Co., Seoul, Korea) for 1 day each at 25 °C to remove organic additives inside the specimens. The weight of the specimens was recorded. The specimens were immersed in toluene for 1 day at room temperature, and the swollen specimens were weighed. The total crosslink density was calculated using the Flory-Rehner equation as follows:

(2) $$\nu =\frac{1}{2M_{c}}=-\frac{\ln\left(1-V_{r}\right)+V_{r}+\chi V_{r}^{2}}{2\rho_{r}V_{s}\left(V_{r}^{\frac{1}{3}}-0.5V_{r}\right)},$$

where *ν* is the crosslink density (mol/g), *M_c_* is the average molecular weight between crosslink points (g/mol), *V_r_* is the volume fraction of rubber in the swollen gel at equilibrium, *Vs* is the molar volume of solvent (cm^3^/mol), *ρ_r_* is the density of rubber sample (g/cm^3^), and *χ* is the polymer-solvent interaction parameter. Further, the chemical crosslink density of the unfilled compounds was calculated based on the Flory–Rehner equation (Equation (2)) and the Kraus equation (Equation (3)):

(3) $$\frac{V_{r0}}{V_{r}}=1-m\left(\frac{\varphi }{1-\varphi }\right),\;$$

where *V_r0_* is the volume fraction of unfilled rubber in the swollen gel at equilibrium, *V_r_* is the volume fraction of rubber in the swollen gel at equilibrium, and *φ* is the volume fraction of filler_._ Subsequently, the filler–rubber interaction was calculated as the difference between the total crosslink density (chemical crosslink density + filler–rubber interaction) and chemical crosslink density.

#### 2.3.8. Mechanical Properties

The mechanical properties of the vulcanizates, including tensile strength, modulus, and elongation at break, were evaluated. Dumbbell-shaped specimens (length = 100 mm; width = 25 mm) were tested at the speed of 500 mm/min using a universal testing machine (UTM, KSU-05M-C, KSU Co., Ansan, Korea) according to the standard procedure given in ATSM D 412.

#### 2.3.9. Abrasion Resistance

Abrasion resistance was measured using an abrasion tester (DIN: Deutsche Industrie Normen, KSU Co., Ansan, Korea) according to the standard procedure given in DIN 53516. Cylindrical specimens (diameter = 16 mm; length = 8 mm) were tested, where an abrasive sheet was rotated on the surface of the specimen at 40 ± 1 rpm under load of 5 N. The mass loss was measured.

#### 2.3.10. Viscoelastic Properties

The viscoelastic properties of the compounds was evaluated by measuring tan δ within a strain range of 0.5 to 10% at 60 °C and 10 Hz using a dynamic mechanical thermal spectrometer (DMTS, EPLEXOR 500N, GABO, Ahlden, Germany). Further, the storage modulus (E′), loss modulus (E″), and tan δ were measured between −70 and 80 °C at 0.2% strain and 10 Hz using a dynamic mechanical analyzer (DMA Q800, TA Instrument, New Castle, DE, USA).

## 3. Results and Discussion

### 3.1. Characterization of Rubber

**Figure 1:**
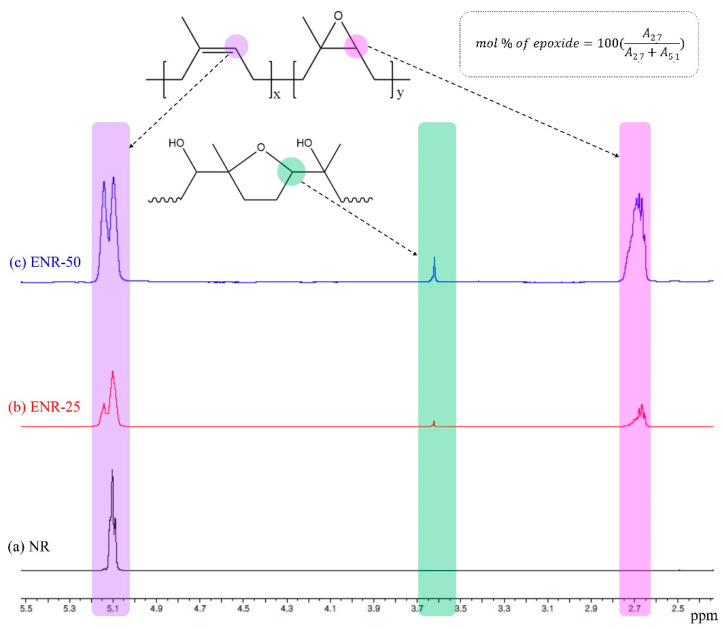
^1^H NMR spectra of (**a**) NR, (**b**) ENR-25, and (**c**) ENR-50.

The ^1^H NMR spectra of NR, ENR-25, and ENR-50 exhibited peaks at 5.1 ppm due to olefinic methine protons, while only ENR-25 and ENR-50 exhibited peaks at 2.7 ppm due to epoxy methine protons (Figure 1) [33,34]. Further, signals were observed at 3.6 ppm in the spectra of ENR-25 and ENR-50 due to the five cyclic ether structure generated due to the ring opening of the adjacent epoxide groups [35]. The epoxide content of the rubber samples was calculated based on the area of the peaks at 5.1 and 2.7 ppm [33,34].

The T_g_ values of NR, ENR-25 and ENR-50 increased linearly as the epoxide contents increased due to the limitation of chain mobility and the interaction between the epoxide groups (Table 3) [33,36]. Specifically, the T_g_ of ENR increased by 0.75 to 0.81 °C per 1 mol% epoxide content. Thus, T_g_ of rubber increased from −61.5 to −25.2 °C as the epoxide contents 46.2 mol% increased.

### 3.2. Payne Effect

**Figure 2:**
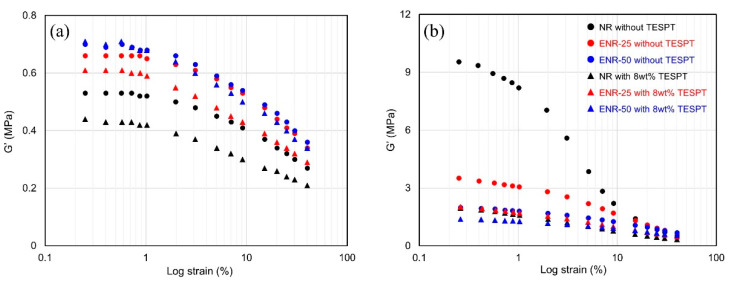
Effect of strain on the shear storage modulus (G′) of the compounds after the first mixing stage: (**a**) before and (**b**) after annealing.

The Payne effect illustrated in Figure 2 and Table 4 provided an indication of the degree of filler–filler interaction and, in turn, the dispersion of uncured compounds [37]. In general, the storage modulus (G′) decreased with increasing strain due to the destruction of the filler–filler network, thus ΔG′ increased as the filler–filler interaction became stronger. However, the hydrogen bonding between the ENRs or between ENR and silica due to ring opening of the epoxide group in the silica-filled ENR compounds were susceptible to damage as strain increased. Thus, the ΔG′ value also increased as the epoxide content of the ENR increased [38,39]. Consequently, the compounds were annealed for 5 min at 160 °C to induce silica re-agglomeration, thereby facilitating examination of the silica dispersion. The Payne effect measured after annealing was increased due to the re-agglomeration of silica, where the ΔG′ values increased as the hydrophobation of silica became more unfavorable. Thus, TESPT improved the hydrophobation of silica in all of the compounds, and silica dispersion improved with increasing epoxide content, as illustrated by the decrease in ΔG′ value.

### 3.3. Cure Characteristics and Mooney Viscosity

**Figure 3:**
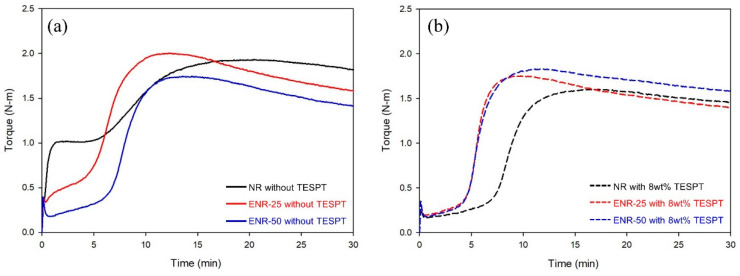
Vulcanization response of the compounds measured by MDR at 150 °C: (**a**) without TESPT and (**b**) with 8 wt% TESPT.

The results of cure characteristics, Mooney viscosity, and bound rubber are shown in Figure 3 and Table 5. Among the compounds without TESPT, the NR compound had a high T_min_ and Mooney viscosity due to very unfavorable silica dispersion, while the ENR compounds had low T_min_ and Mooney viscosity due to excellent silica dispersion, even without TESPT, as the epoxide content increased. The compounds with 8 wt% TESPT had lower Mooney viscosity and T_min_ compared to those without TESPT due to improved silica dispersion. The bound rubber contents of these samples were also lower due to a decrease in occluded rubber [40]. The Mooney viscosity of the ENR compounds was higher than the NR compounds due to the higher bound rubber contents of the epoxide groups, where basic silica dispersion was achieved without the addition of TESPT. Furthermore, the increased crosslink density due to the epoxide groups led to higher ∆T values, and the ENR compounds had faster t_10_ and t_90_ values than the NR compounds due to the lower activation energy of the double bonds adjacent to the epoxide groups [41].

### 3.4. Crosslink Density and Vulcanizate Structure Analysis

**Figure 4:**
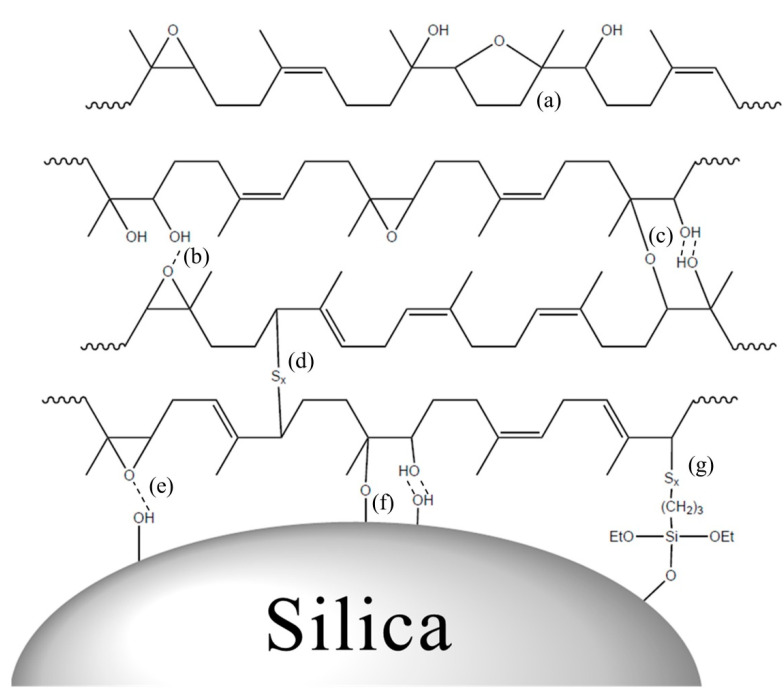
Proposed interaction mechanism of silica filled ENR vulcanizates: (**a**) ring opening of adjacent epoxide groups to yield five membered cyclic ether; (**b**) epoxide group ring opening and hydrogen bonding between epoxide group and hydroxyl group; (**c**) self-crosslinking of ENR via ring-opened epoxide groups; (**d**) chemical crosslinking by sulfur; (**e**) hydrogen bonding between silica and ENR; (**f**) direct silica-epoxide coupling, and (**g**) silica-TESPT-rubber interaction.

**Figure 5:**
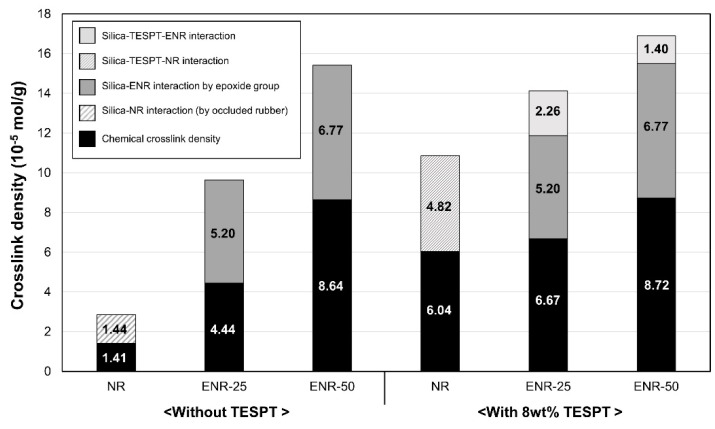
Crosslink densities of the vulcanizates without TESPT and with 8 wt% TESPT.

The interactions and chemical bonding in the silica filled ENR vulcanizates are illustrated in Figure 4. Hydrogen bonding occurred in ENR between the hydroxyl and epoxide groups (Figure 4b) due to ring-opening of the epoxide groups, while self-crosslinking also occurred (Figure 4c) [18,42,43,44]. Further, five membered cyclic ether structures could be formed if the epoxide group was repeated (Figure 4c) [13,14,44,45]. Hydrogen bonding occurred between ENR and silica (Figure 4e), and direct silica–epoxide coupling occurred between the hydroxyl group of silica and epoxide group of ENR (Figure 4f). Silica–TESPT–rubber interaction was possible when a silane coupling agent, such as TESPT, was employed (Figure 4g). Chemical crosslinking due to sulfur also occurred (Figure 4c). Thus, the silica-filled ENR compound vulcanizates had complex vulcanizate structures, which were analyzed based on the total crosslink density (Figure 5 and Table 6). This was calculated as the sum of the filler–rubber interaction and chemical crosslink density based on a vulcanizate structure analysis of the compounds with different filler contents [24,25,26,27,28,29,30,31,32]. When ENR was used as the base rubber, the silica–TESPT–ENR interactions and silica–ENR interactions due to the epoxide groups were quantitatively calculated based on the difference in filler–rubber interactions between the compounds with and without 8 wt% TESPT.

The NR compound without TESPT exhibited little filler–rubber interaction due to the influence of occluded rubber caused by the very unfavorable silica dispersion [24]. However, this influence of occluded rubber was decreased in the ENR compounds without TESPT due to the improved silica dispersion achieved by the presence of epoxide groups. Thus, the filler–rubber interaction was increased due to the interaction between the epoxide groups and silica. As reflected in the Payne effect, the NR compounds without TESPT exhibited a low chemical crosslink density due to adsorption of the accelerators by the silica, which was attributed to its low level of hydrophobation. The chemical crosslink density of the ENRs was increased due to interaction between the ENRs, such as hydrogen bonding and self-crosslinking due to ring opening of the epoxide group, as well as reduced accelerator adsorption of silica with improved hydrophobation after epoxidation [46].

The compounds with 8 wt% TESPT exhibited higher crosslink densities than those without TESPT due to reduced accelerator adsorption as the hydrophobation of silica was improved in all compounds. Filler–rubber interactions also increased due to silica–TESPT–rubber interactions induced by the coupling reaction of TESPT. The differences in filler–rubber interactions between the compounds with and without 8 wt% TESPT were used to evaluate the silica–ENR interactions facilitated by the epoxide groups, as well as the silica–TESPT–ENR interactions. The silica–TESPT–rubber interactions decreased and silica–ENR interactions by the epoxide groups increased substantially as the epoxide content increased. This demonstrated that TESPT can be partially replaced by introducing epoxide groups to NR to facilitate higher filler–rubber interaction.

### 3.5. Mechanical Properties and Abrasion Resistance

**Figure 6:**
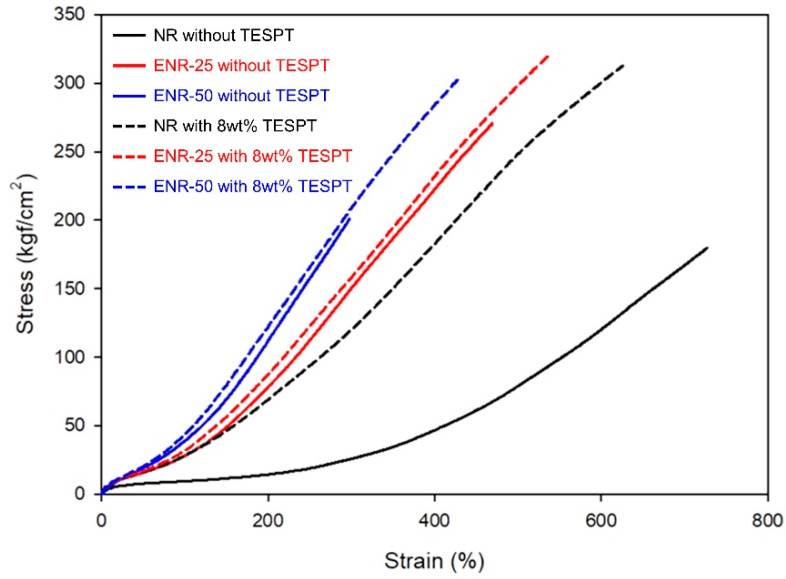
Stress-strain curves of the vulcanizates.

The mechanical properties of the various silica filled compounds are presented in Figure 6 and Table 7. The stress–strain curves indicated that the NR compound without TESPT had a low modulus due to its low crosslink density, whereas the ENR compounds without TESPT had a high modulus due to the improved crosslink density with increasing epoxide content. The compounds with 8 wt% TESPT exhibited an increase in modulus compared to the compounds without TESPT due to increased crosslink density. Further, the elongation at break increased due to the improved silica dispersion.

**Figure 7:**
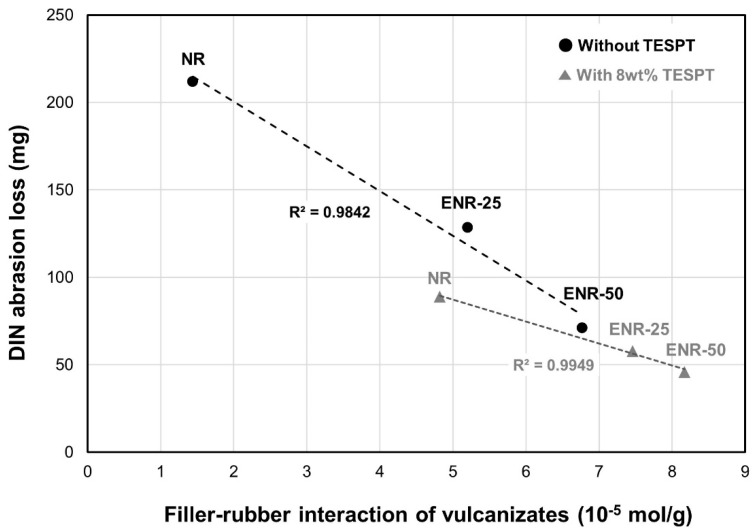
Effect of vulcanizate filler–rubber interaction on DIN abrasion loss.

Abrasion resistance was closely related to the filler–rubber interactions of the vulcanizates (Figure 7). The compounds without TESPT exhibited significantly improved abrasion resistance as the epoxide content increased due the enhanced silica dispersion and the filler–rubber interaction. Further, the compounds with 8 wt% TESPT exhibited superior abrasion resistance compared to those without TESPT. Thus, the enhancement of filler–rubber interaction due to increased epoxide content facilitated improvements in abrasion resistance, where DIN abrasion resistance had a linear relationship with the filler–rubber interaction of the vulcanizates.

### 3.6. Dynamic Viscoelastic Properties

**Figure 8:**
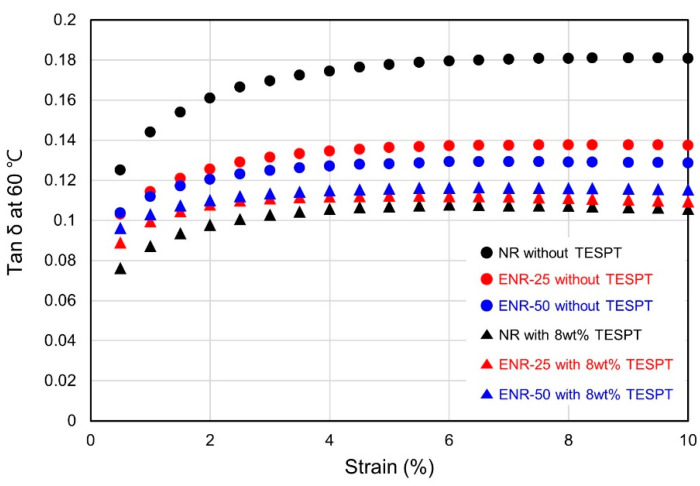
Effect of strain on the tan δ values of the vulcanizates at 60 °C.

The rolling resistance (RR) of tires can be evaluated by assessing the dynamic viscoelastic properties and determining tan δ at 60 °C, where a lower value is indicative of better fuel efficiency [47]. Further, the loss modulus (E″) at 0 °C is an indicator of the wet grip performance of tires, where a higher value is indicative of improved wet grip performance [48,49]. Tan δ values were measured at varying strains, at 60 °C via DMTS (Figure 8 and Table 8). In the compounds without TESPT, with increasing epoxide content, the tan δ values at 60 °C were low due to improved silica dispersion and increased crosslink density. However, the compounds with 8 wt% TESPT were not significantly affected by silica dispersion according to epoxidation, as the essential silica dispersion was achieved by TESPT. This led to high tan δ values at 60 °C as the epoxide content increased.

**Figure 9:**
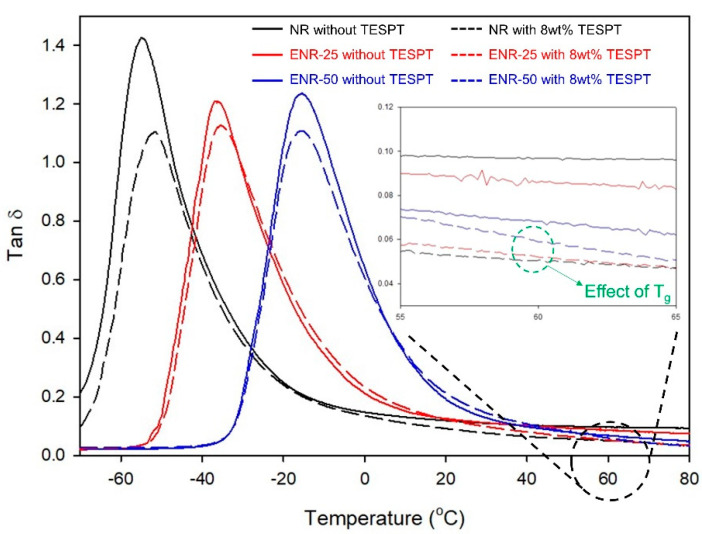
Effect of temperature on the tan δ value of the vulcanizates at 0.2% strain.

Rubber compounds tend to exhibit a high tan δ value near T_g_ due to hysteresis of the rubber chain, and the T_g_ of a rubber compound is considerably affected by the T_g_ of its base rubber [8]. The T_g_ of ENR is high, and increases with increasing epoxide content. Therefore, the T_g_ of the ENR compounds were expected to increase as the epoxide content increased. The effects of T_g_ on the tan δ values of the compounds at 60 °C were evaluated using DMA analysis over a temperature sweep (Figure 9 and Table 9). The increase in T_g_ of the compounds with increasing epoxide content was confirmed, and tan δ at T_g_ decreased as the hysteresis of the rubber chain decreased due to the improved crosslink density in the compounds with 8 wt% TESPT compared to those without [50,51]. Following the trend of the DMTS measurements, the tan δ values at 60 °C decreased as silica dispersion improved with increasing epoxide content in the compounds without TESPT. However, the compounds with 8 wt% TESPT exhibited high tan δ values at 60 °C due to the increase in T_g_ with increasing epoxide content, which had a larger impact than the beneficial improvement in silica dispersion [18]. In contrast, the E″ at 0 °C was evaluated to assess the wet grip performance, where values increased as T_g_ approached 0 °C. This was indicative of excellent wet grip performance as the epoxide content increased, which was substantially improved in the ENR-50 compounds.

## 4. Conclusions

The requirements for TBR tire treads are continually increasing to achieve outstanding fuel efficiency. Recent research has focused on replacing carbon black with silica. Therefore, this study used ENR as the base rubber to resolve issues related to coupling reactions between silica and rubber, and unfavorable silanization reactions in silica-filled NR compounds. The filler–rubber interactions were evaluated with respect to the presence of TESPT, where silica-ENR interactions via the epoxide groups and silica–TESPT–rubber interaction due to TESPT were quantitatively calculated during vulcanizate structure analysis. This allowed for analysis of the vulcanizate structure of the silica-filled ENR vulcanizates.

Among the compounds without TESPT, the NR compound exhibited very unfavorable silica dispersion, whereas the ENR compounds without TESPT had improved silica dispersion as the epoxide content increased. Further, abrasion resistance was also significantly improved by epoxidation due to the enhanced filler–rubber interactions, while the fuel efficiency was also improved due to the increased crosslink density and enhanced silica dispersion. All of the compounds with 8 wt% TESPT exhibited superior silica dispersion compared to those without TESPT, where the filler–rubber interaction was increased due to silica–TESPT–rubber interactions. Thus, both the abrasion resistance and fuel efficiency were enhanced. In the compounds with 8 wt% TESPT, similar to the trend observed for the compounds without TESPT, where excellent abrasion resistance was achieved as epoxide content increased due to high filler–rubber interaction. However, the tan δ values at 60 °C were high due to the influence of the T_g_ of the compounds, thereby leading to a slight compromise in fuel efficiency. The E″ value at 0 °C was high as T_g_ increased, which was indicative of an outstanding wet grip performance.

The vulcanizate structure analysis demonstrated that more silica-ENR interaction was achieved due to the presence of epoxide groups compared to the silica-TESPT-rubber interactions facilitated by TESPT. This confirmed that the epoxidation of NR can lead to higher filler–rubber interactions than the addition of TESPT. Therefore, the epoxidation of NR resolves issues such as low filler–rubber interaction due to unfavorable coupling reactions and silanization reactions of silica-filled NR compounds. Further, the abrasion resistance can be improved by enhancing the filler–rubber interaction as the epoxide content increases. Thus, this study quantitatively confirms that the epoxidation of NR can solve the common issues associated with silica-filled NR compounds.

Therefore, through this study, it will be possible to understand the complex vulcanizate structure of silica filled ENR compounds, and to develop a TBR tire tread with excellent wear resistance, fuel efficiency and wet grip.

## Figures and Tables

**Table 1:** Composition of the compounds (phr).

	Compounds without TESPT			Compounds with 8 wt% TESPT *		
	NR	ENR-25	ENR-50	NR	ENR-25	ENR-50
NR	100	0	0	100	0	0
ENR-25	0	100	0	0	100	0
ENR-50	0	0	100	0	0	100
Silica	55	55	55	55	55	55
TESPT *	0	0	0	4.4	4.4	4.4
ZnO	4	4	4	4	4	4
Stearic acid	3	3	3	3	3	3
6PPD	2	2	2	2	2	2
TMQ	1	1	1	1	1	1
Sulfur	1.5	1.5	1.5	1.5	1.5	1.5
CBS	1.5	1.5	1.5	1.5	1.5	1.5
DPG	1.06	1.06	1.06	1.06	1.06	1.06
PVI	0.3	0.3	0.3	0.3	0.3	0.3

* The amount of silane coupling agents was calculated as 8 wt% of the weight of silica.

**Table 2:** Mixing procedure used to manufacture the compounds and vulcanizates.

	Time (min:s)	Action
1st Stage	0:00–1:30	NR or ENR mastication (initial temp.: 100 °C)
	1:30–2:40	Add half of the silica and half of the silane
	2:40–3:40	Add remaining silica and silane
	3:40–5:30	Add ZnO, St/A, 6PPD, and TMQ
	5:30	Ramp up
	5:30–7:30	Extra mixing and dump (dump temp.: 150–155 °C)
2nd Stage	0:00–0:30	Master batch from first stage (initial temp.: 50 °C)
	0:30–2:30	Curatives and dump (dump temp.: 80–90 °C)

**Table 3:** Epoxide contents and T_g_ of rubber determined using ^1^H NMR and DSC.

Rubber Sample	Epoxide Content (%)	Tg (°C)
NR	0	−61.5
ENR-25	25.3	−42.2
ENR-50	46.2	−25.2

**Table 4:** Payne effect based on ΔG′ (0.28–40.04%, MPa) before and after annealing.

	Without TESPT			With 8 wt% TESPT		
	NR	ENR-25	ENR-50	NR	ENR-25	ENR-50
Before annealing	0.26	0.32	0.34	0.23	0.32	0.37
After annealing *	9.01	2.85	1.30	1.62	1.55	0.84

* Annealing conditions: 160 °C, 5 min.

**Table 5:** Cure characteristics and Mooney viscosity of the compounds.

	Without TESPT			With 8 wt% TESPT		
	NR	ENR-25	ENR-50	NR	ENR-25	ENR-50
Mooney viscosity (ML_1+4_ at 100 °C)	90.2	85.6	74.3	61.5	72.3	72.0
Bound rubber content (%)	38.6	53.0	54.5	37.5	47.4	50.8
T_min_ (N-m)	1.00	0.34	0.18	0.17	0.20	0.18
T_max_ (N-m)	1.93	2.00	1.74	1.60	1.75	1.83
ΔT (N-m)	0.93	1.66	1.58	1.43	1.55	1.65
t_10_ (min:s)	6:09	2:14	5:20	6:11	4:10	4:15
t_90_ (min:s)	13:49	8:51	10:12	11:21	6:54	7:45

**Table 6:** Crosslink densities of the vulcanizates (10^−5^ mol/g).

		Without TESPT			With 8 wt% TESPT		
		NR	ENR-25	ENR-50	NR	ENR-25	ENR-50
Total crosslink density (chemical crosslinking + filler–rubber interaction)		2.85	9.65	15.41	10.85	14.13	16.89
Chemical crosslink density		1.41	4.44	8.64	6.04	6.67	8.72
Filler–rubber interaction:	Silica–NR interaction (by occluded rubber)	1.44	-	-	-	-	-
	Silica–TESPT–NR interaction	-	-	-	4.82	-	-
	Silica–ENR interaction by epoxide group	-	5.20	6.77	-	5.20	6.77
	Silica–TESPT–ENR interaction	-	-	-	-	2.26	1.40

**Table 7:** Mechanical properties and DIN abrasion loss of the vulcanizates.

	Without TESPT			With 8 wt% TESPT		
	NR	ENR-25	ENR-50	NR	ENR-25	ENR-50
M_100%_ (kgf/cm^2^)	9.5	28.1	38.6	28.3	31.9	43.4
M_300%_ (kgf/cm^2^)	25.9	150.1	-	119.6	157.8	208.7
Elongation at break (%)	730	470	298	630	540	430
Tensile strength (kgf/cm^2^)	180	271	201	313	321	312
DIN abrasion loss (mg)	211.8	128.5	71.3	89.0	58.0	46.0

**Table 8:** Tan δ values of the vulcanizates at 60 °C and 5% strain.

Without TESPT			With 8 wt% TESPT		
NR	ENR-25	ENR-50	NR	ENR-25	ENR-50
0.178	0.136	0.128	0.107	0.112	0.116

**Table 9:** Viscoelastic properties of the vulcanizates at 0.2% strain.

	Without TESPT			With 8 wt% TESPT		
	NR	ENR-25	ENR-50	NR	ENR-25	ENR-50
T_g_ (°C)	−54.8	−36.2	−15.4	−51.6	−35.5	−15.7
E″ at 0℃ (MPa)	1.05	2.67	14.55	2.11	3.44	15.87
Tan δ at 60 °C	0.097	0.086	0.068	0.051	0.052	0.059

## Data Availability

Data presented in this study are available on request from the corresponding author.
